# Metabolic reprogramming in hepatocellular carcinoma: mechanisms and therapeutic implications

**DOI:** 10.1038/s12276-025-01415-2

**Published:** 2025-03-03

**Authors:** Sujin Park, Michael N. Hall

**Affiliations:** 1 Center for Genome Engineering, Institute for Basic Science, Daejeon, Republic of Korea; 2 Biozentrum, University of Basel, Basel, Switzerland

**Keywords:** Cancer metabolism, Cancer therapy

## Abstract

Hepatocellular carcinoma features extensive metabolic reprogramming. This includes alterations in major biochemical pathways such as glycolysis, the pentose phosphate pathway, amino acid metabolism and fatty acid metabolism. Moreover, there is a complex interplay among these altered pathways, particularly involving acetyl-CoA (coenzyme-A) metabolism and redox homeostasis, which in turn influences reprogramming of other metabolic pathways. Understanding these metabolic changes and their interactions with cellular signaling pathways offers potential strategies for the targeted treatment of hepatocellular carcinoma and improved patient outcomes. This review explores the specific metabolic alterations observed in hepatocellular carcinoma and highlights their roles in the progression of the disease.

## Glycolysis shift fuels liver cancer growth and resistance

Cancer cells often change their metabolism to support rapid growth. This study examines how liver cancer, specifically hepatocellular carcinoma (HCC), alters its metabolism and explores potential treatments. The authors focus on how HCC cells use glucose, amino acids and fatty acids differently from normal cells. HCC cells rely heavily on glycolysis even when oxygen is present, a phenomenon known as the Warburg effect. They also activate the pentose phosphate pathway to produce molecules needed for growth and survival. Additionally, HCC cells depend on certain amino acids such as glutamine and serine for building blocks and energy. This study highlights that targeting these altered metabolic pathways could be a promising strategy for treating HCC. In conclusion, understanding these metabolic changes in HCC can lead to new treatment approaches.

This summary was initially drafted using artificial intelligence, then revised and fact-checked by the author.

## Introduction

Metabolic rewiring in cancer entails adaptive modifications to meet specific demands imposed by enhanced proliferation. This rewiring encompasses both anabolic and catabolic pathways, enabling cells to maintain uncontrolled replication and, ultimately, survival^1^.

The liver, as the primary metabolic organ, plays an important role in regulating metabolic processes essential for overall homeostasis. These include glucose, protein and lipid metabolism, which impact energy balance regulation, nutrient storage, detoxification and the synthesis of vital biomolecules^2–4^. Hepatocellular carcinoma (HCC), often detected late, presents diagnostic challenges due to it frequently occurring alongside pre-existing liver conditions such as cirrhosis, chronic hepatitis B or C infection, or metabolic dysfunction-associated fatty liver disease. This complicates treatment and limits therapeutic options. Moreover, HCC exhibits resistance to certain chemotherapeutic agents and is prone to high recurrence rates post-treatment. The liver’s unique metabolic and detoxifying capabilities also influence the pharmacokinetics of drugs, further complicating treatment. Despite the FDA approval of the first tyrosine kinase inhibitor (TKI) for HCC treatment, resistance develops rapidly in many patients^5,6^. Similarly, other TKIs have shown limited survival benefits^7,8^. Nivolumab, an FDA-approved immune checkpoint inhibitor for second-line treatment, has shown limited efficacy in patients with HCC, further underscoring the urgent need for better therapeutic strategies^9^. Additionally, the liver’s susceptibility to metastases from primary cancers in the gastrointestinal tract poses further challenges^10,11^. This review provides current insights into cancer metabolism, specifically within the liver, and discusses potential therapeutic approaches that exploit metabolic pathways altered in HCC to inhibit tumor growth.

## Main

### Enhancement of glycolysis

**Fig. 1:**
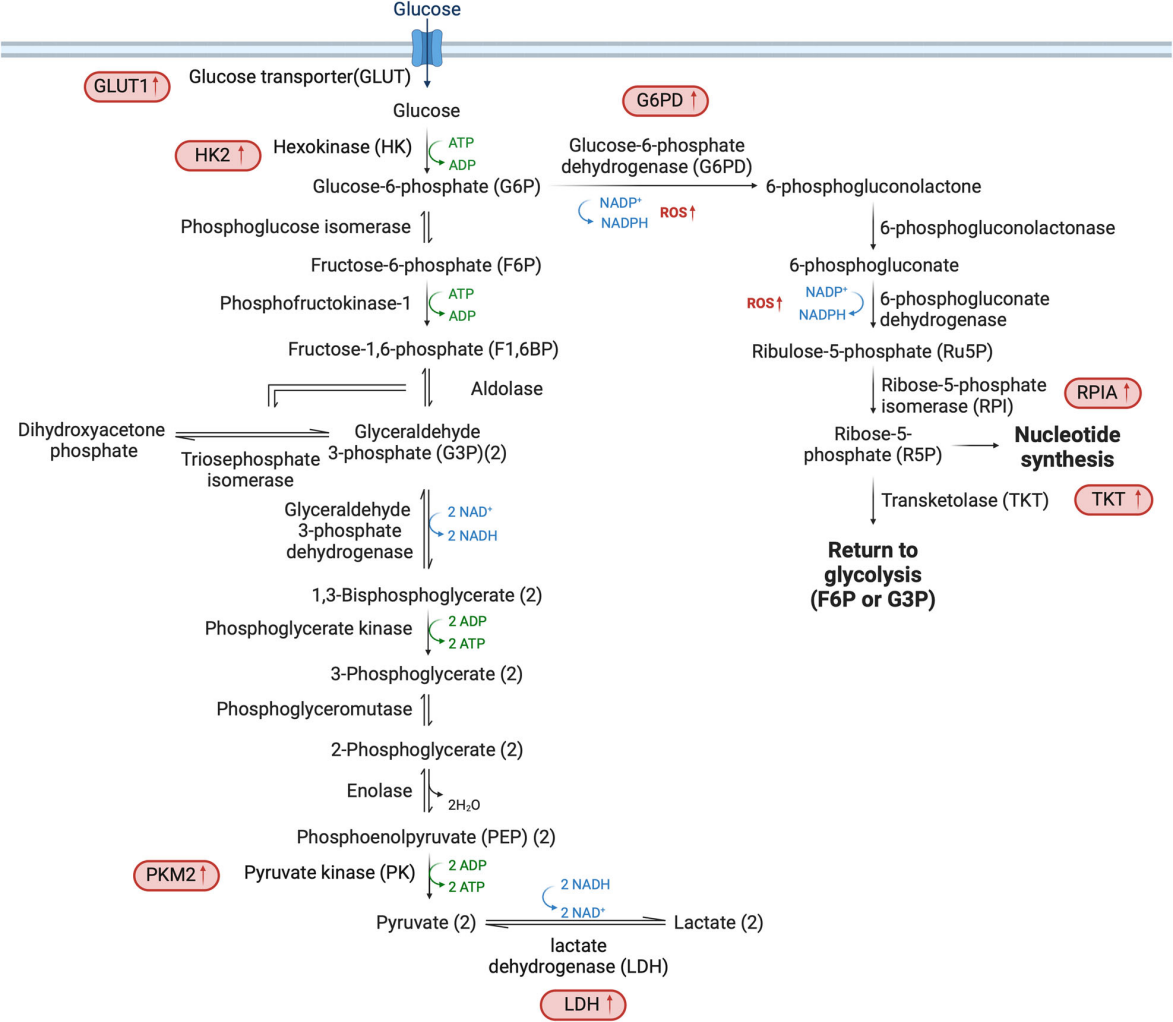
Rewired glycolysis and pentose phosphate pathway in HCC. The significant shift in glucose metabolism within HCC cells, characterized by upregulated expression of key enzymes that enhance glucose uptake and utilization. These changes include increased levels of GLUT1 and HK2, which elevate glucose uptake; PKM2, which increases augments pyruvate formation; and LDH, which facilitates the conversion of pyruvate to lactate, supporting anaerobic glycolysis. The diagram also highlights the activation of the PPP, a crucial metabolic pathway that provides R5P for nucleotide synthesis and NADPH for lipid synthesis and antioxidant defense. In HCC, the PPP is often enhanced, as indicated by increased activity of enzymes such as G6PD and TKT. Enzymes with increased expression in HCC are marked in red, denoting their upregulation and pivotal roles in cancer metabolism.

The liver plays a central role in the regulation of blood glucose levels. The body stores glucose in the form of glycogen during periods of surplus and subsequently releases glucose into the circulatory system when blood glucose levels decrease, thereby mediating glucose homeostasis. During periods of fasting or heightened energy requirements, the liver releases glucose via glycogen catabolism^4^. In HCC, there is a marked reliance on glycolysis for energy production, even in the presence of oxygen, a phenomenon known as the Warburg effect^12^. This shift from oxidative phosphorylation to glycolysis allows HCC cells to efficiently produce energy in oxygen-rich environments, typical of anaerobic metabolism. Key glycolytic genes, including those encoding glucose transporters (GLUTs), hexokinase (HK) and pyruvate kinase (PK), are differentially expressed in HCC, leading to increased glucose uptake and metabolism (Fig. 1).

The GLUT1–4 family of transporters enables the uptake of glucose across the plasma membrane into the cytoplasm^13^. The comparative analysis of GLUT1 (refs. ^14^^[,15^) and GLUT2 (ref. ^16^) expression in HCC and surrounding tissue reveals a correlation of elevated GLUT1 levels with poor prognosis^15^. Upon entering the cell, glucose undergoes an irreversible conversion to glucose-6-phosphate (G6P), resulting in a significant increase in the levels of G6P in HCC tumors compared with the surrounding liver tissue^17^. This conversion is facilitated by a group of enzymes referred to as HKs. HK4 is the HK isoform normally expressed in hepatocytes^18^, but HK2 is often upregulated in HCC and is closely linked to the clinical stage of the disease and the prognosis of patients^19,20^. The enhanced ability of HK2 to boost aerobic glycolysis, compared with other HK isoforms, can be attributed to its binding the voltage-dependent anion-selective channel protein-1 located in the mitochondrial membrane. This interaction increases access to mitochondrially generated ATP^21,22^. Thus, hepatic HK2 deletion inhibits tumorigenesis and increases cell death both in vitro and in vivo^19,21^. The last enzyme in glycolysis is PK, which transfers phosphate from phosphoenolpyruvate (PEP) to ADP to yield ATP and pyruvate. The M2 isoform of PK (PKM2) is observed mainly in rapidly proliferating cells^23^ and has been correlated with tumor formation in HCC^1,24,25^. The conversion of pyruvate to lactate is facilitated by lactate dehydrogenase (LDH), and elevated levels of LDH^26^ in the bloodstream have been linked to poor progression-free survival in patients with HCC^27^. Indeed, high levels of LDH and secreted lactate are common features of HCC.

Transcriptomic and metabolomic studies in HCC have shown depletion of major metabolites such as glucose, glycerol 3- and 2-phosphate and malate, alongside an increase in glycolytic activity over mitochondrial oxidative phosphorylation^28^. The upregulation of glycolytic enzymes is often driven by aberrant activation of the Wnt/β-catenin and PI3K/Akt/mTOR pathways, which are pivotal in HCC’s altered glucose metabolism^29,30^. Additionally, the expression of the glucose-responsive transcription factor carbohydrate responsive element-binding protein (ChREBP) is elevated in HCC and correlates with tumor aggressiveness. ChREBP is a key regulator of glycolysis, the pentose phosphate pathway and lipogenic genes^31^. Therefore, given its crucial role in hepatic energy metabolism, ChREBP may represent a promising target for therapeutic intervention in HCC.

Targeting glycolysis has therapeutic potential^32,33^, as evidenced by strategies that disrupt HK2 function^34^ or inhibit other glycolytic enzymes^35–38^. The hypoxic tumor microenvironment and resulting induction of expression of GLUT1, LDH and hypoxia-inducible factor-1 alpha, further exemplifies metabolic adaptation in HCC^6,14,39^. In conclusion, HCC displays reprogrammed glycolysis, which is essential for tumorigenesis.

### Activation of the pentose phosphate pathway

The pentose phosphate pathway (PPP) is crucial for cancer cell proliferation. Oncogenic metabolic reprogramming encompasses an increase of metabolic flow through the PPP to enhance the supply of nucleotides and reducing equivalents^40^. In particular, increased PPP diverts G6P to produce ribose 5-phosphate (R5P)^41^ and NADPH. R5P is essential for nucleotide synthesis, while NADPH plays a key role in lipid synthesis and acts as a major antioxidant by managing cellular levels of reactive oxygen species (ROS).

G6P dehydrogenase (G6PD), the rate-limiting enzyme of oxidative PPP, catalyzes the conversion of G6P to 6-phosphogluconolactone^42^. In HCC, G6PD is often upregulated^43,44^, enhancing aspects of malignancy such as apoptosis resistance, migration, invasion and the epithelial–mesenchymal transition^45–47^. The underlying mechanism by which G6PD expression and activity are controlled in HCC remains poorly understood. In clinical settings, the upregulation of G6PD expression has been observed to promote chemoresistance in HCC cells. This can be attributed to the activation of the ID1–WNT–β-catenin–MYC signaling pathway^45^. In addition, mTORC1 enhances the flux of substances through PPP by facilitating glycolysis, which provides substrates for PPP, and by elevating the levels of G6PD and R5P isomerase A^48^. The action of nuclear factor-erythroid 2-related factor 2 (NRF2) is also crucial for the expression of G6PD. Mice lacking NRF2 demonstrate resistance to HCC induced by diethylnitrosamine as a result of decreased production of enzymes associated with PPP^49^.

Transketolase (TKT) is also a critical PPP enzyme. TKT’s activity is regulated by competition between the transcription factors NRF2 and BACH1, with NRF2 enhancing TKT expression to convert glucose derivatives into glutathione, thereby protecting cells from ROS-induced damage^50^. Elevated TKT levels in HCC are associated with increased tumor aggressiveness^51^ and poor response to treatments with the TKI sorafenib^50^.

The intricate regulation of PPP by factors such as NRF2 and the WNT and mTORC1 pathways suggests that these factors could be targeted to improve patient outcomes in HCC.

### Dependence on specific amino acids

#### Glutamine metabolism

**Fig. 2:**
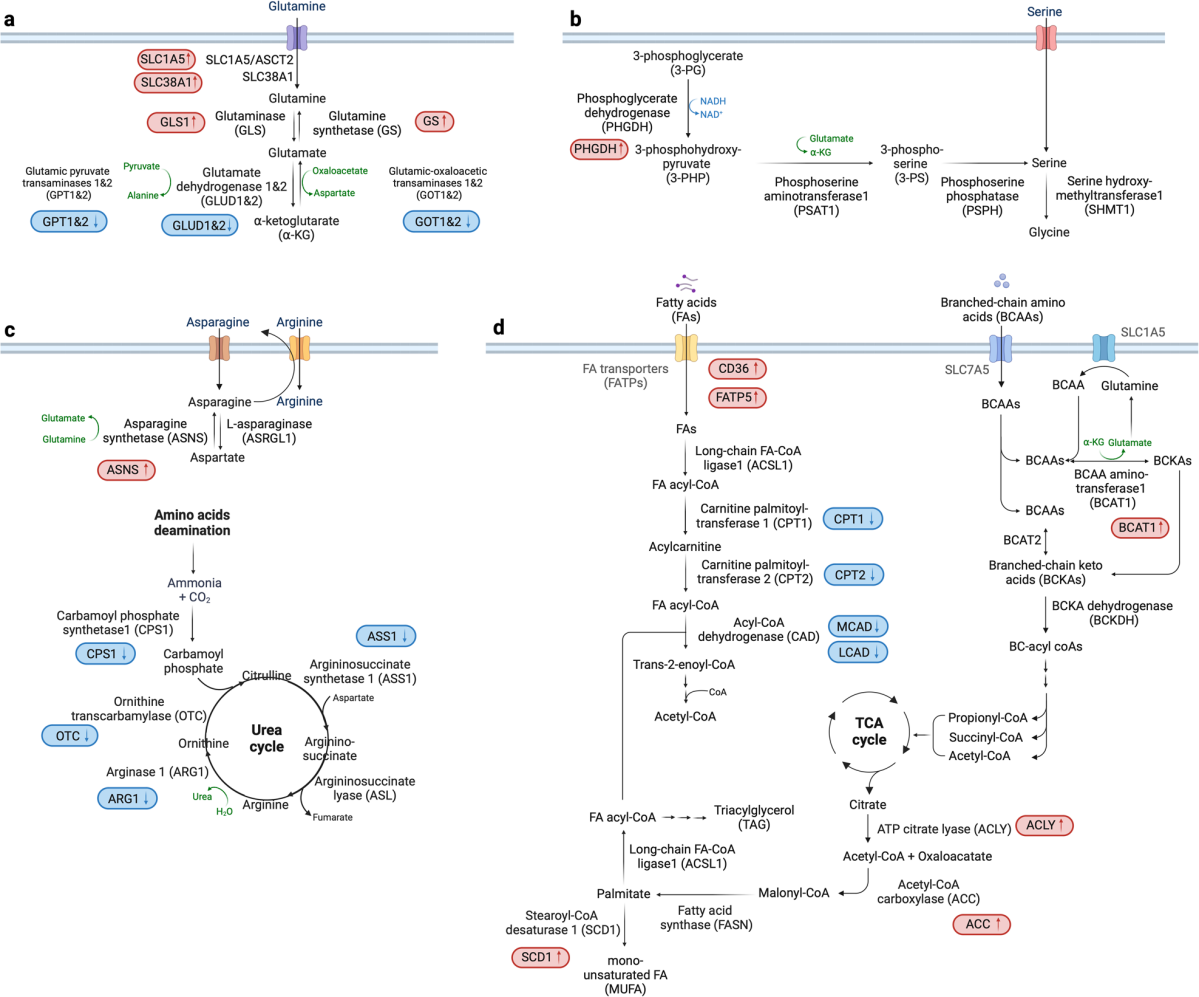
Rewired amino acid metabolism and FA metabolism in HCC. **a**–**d**, The critical dependencies and alterations in amino acid (**a**–**c**) and FA metabolism within HCC cells (**d**). Glutamine is pivotal for nucleotide and amino acid production and antioxidant synthesis. In HCC, enhanced glutamine uptake is facilitated by upregulated transporters such as SLC38A1 and SLC1A5, while the conversion to α-ketoglutarate is suppressed due to downregulation of glutamate dehydrogenase 1 and 2 (GLUD1 and 2), promoting glutaminolysis. Elevated levels of GS are associated with increased cell proliferation, highlighting its complex role in cancer progression (**a**). Serine is essential for nucleotide synthesis and maintaining redox balance, derived from external sources and metabolic pathways from glucose or glutamine. Increased activity of PHGDH boosts de novo serine synthesis in HCC (**b**). The urea cycle and arginine metabolism involve increased ammonia production and disruptions in metabolism leading to elevated nitrogen supply for biosynthesis. The downregulation of enzymes such as CPS1, ASS1, ARG1 and OTC results in abnormal ammonia metabolism, while dysregulated arginine metabolism promotes oncogenic activities (**c**). BCAAs undergo conversion to BCKAs, crucial for nucleotide biosynthesis and energy production via the TCA cycle and linked to FAO pathways. Fatty acid metabolism is characterized by increased de novo synthesis and altered FAO. Key enzymes such as ACLY, ACC, FASN, SCD1 and CPT1 are dysregulated, contributing to the metabolic reprogramming that supports tumor growth and progression (**d**). Proteins with increased levels in HCC are marked in red, while those decreased are shown in blue, providing a clear visual differentiation of metabolic changes in HCC.

The nonessential amino acid glutamine plays a pivotal role in HCC metabolism, serving as a carbon source for anaplerosis and as a nitrogen source for nucleotide and amino acid synthesis^52,53^. It also contributes significantly to other biosynthetic processes, including anti-ROS glutathione/NADPH production and lipid synthesis^54^. Notably, altered glutamine metabolism involves the upregulation of specific transporters and enzymes such as sodium-coupled neutral amino acid transporters (SLC38A1 and SLC1A5/ASCT2) and of glutaminase 1 and 2 (GLS1 and 2), enhancing glutamine uptake and its conversion to alpha-ketoglutarate (α-KG)^55,56^, respectively (Fig. 2a). GLS1 upregulation is particularly pronounced compared with GLS2, which is the isoform typically found in hepatocytes. The glutaminases catalyze the first step in the conversion of glutamine to α-KG, a two-step deamination process. This change in glutaminolysis, often regulated by MYC^57^, correlates with disease progression and has implications for patient prognosis^58^.

Glutamine synthetase (GS), regulated by the Wnt/β-catenin pathway, is another important enzyme in glutamine metabolism. Its level is associated with increased cell proliferation and varied prognostic outcomes^59,60^. Indeed, emerging studies suggest a complex role for GS in HCC^61^, potentially linked to cellular differentiation and tumor behavior^62,63^. Furthermore, the dysregulation of glutamine metabolism impacts several downstream signaling pathways, notably mTORC1 (refs. ^64,65^) and mTORC2–AKT–C-MYC^66^, affecting cellular bioenergetics and contributing to the oncogenic processes in HCC. The hepatocyte growth factor axis also influences these metabolic pathways, underscoring the intricate interplay between growth factor signaling and metabolic reprogramming in cancer progression^67^.

#### Serine metabolism

Serine is an essential amino acid crucial for nucleotide synthesis and redox homeostasis. It is predominantly synthesized de novo from glucose or glutamine metabolites, but can also be obtained from extracellular sources. Notably, studies have shown that serine levels are significantly elevated in the serum of patients with HCC compared with healthy individuals^68^, suggesting altered serine metabolism in the tumor environment.

At the molecular level, the transcription factor NRF2 plays a vital role in HCC by regulating intracellular ROS and enhancing the activity of phosphoglycerate dehydrogenase (PHGDH) (Fig. 2b). PHGDH catalyzes the first step in serine biosynthesis, and its upregulation leads to increased serine synthesis^69^, underscoring the importance of NRF2 in maintaining cellular redox balance and promoting tumor growth. Moreover, hyperactivation of PHGDH and subsequent serine accumulation are associated with poor prognosis in patients with HCC^70^. PHGDH has been implicated as a driver of sorafenib resistance in HCC, and treatment with the PHGDH inhibitor NCT-503 has been shown to synergize with sorafenib, effectively inhibiting HCC growth in vivo^71^. Studies have also indicated that serine is not merely a metabolic byproduct, but a critical substrate in HCC pathophysiology. A deficiency in serine impedes HCC cell growth, highlighting serine metabolism as a vulnerability in cancer cells^69^.

#### Urea cycle, arginine and asparagine metabolism

The urea cycle is fundamental to liver function, converting harmful ammonia, a byproduct of amino acid and protein catabolism, into urea for excretion. Key enzymes in this process include carbamoyl phosphate synthetase 1 (CPS1), argininosuccinate synthetase 1 (ASS1), argininosuccinate lyase (ASL), arginase 1 (ARG1) and ornithine transcarbamylase (OTC)^72,73^. In HCC, disruptions in this cycle contribute to abnormal ammonia metabolism and an increased availability of nitrogen for nucleotide synthesis, supporting rapid tumor growth (Fig. 2c).

HCC is characterized by epigenetic downregulation of CPS1 and ASS1 through hypermethylation^74^, impacting cell proliferation and apoptosis. In particular, ASS1 suppression leads to arginine autotrophy^74,75^ that enhances tumor cell migration, invasion and metastasis^76^. ARG1 catalyzes the breakdown of arginine into ornithine and urea. Ornithine then undergoes additional metabolism to form polyamines, which mediate numerous cellular processes^75,77^. ARG1 is the predominant isoform in the hepatic urea cycle, whereas ARG2 is expressed mainly in extrahepatic tissues^78^. Arginine, an amino acid that is considered conditionally essential, plays a role in various biological processes and has been associated with tumorigenesis^79^. Arginine is taken up into the cell mainly via SLC7A1, and HCC shows a high amount of intracellular arginine^80,81^. The arginine mediates further metabolic reprogramming by binding directly to the transcription co-factor RBM39 (ref. ^82^). It is noteworthy that the synthesis of asparagine exhibits a strong correlation with arginine uptake in HCC^82^, probably due to the use of asparagine as an anti-solute in arginine import.

The synthesis of asparagine occurs through an ATP-dependent process catalyzed by asparagine synthetase (ASNS). Glutamic acid is utilized as the nitrogen source in this process^83^. The expression of ASNS is increased in HCC tumors and is associated with the tumor stage and prognosis. Suppression of ASNS hinders the proliferation, migration and development of HCC cells, indicating its potential as a target for therapeutic intervention^84^. Furthermore, ARG1 expression is substantially decreased in HCC. The combined effects of increased asparagine-dependent arginine uptake and decreased ARG1-dependent arginine conversion to polyamines account for the elevated levels of arginine in liver cancer cells. This metabolic rewiring further mediates the development of oncogenic metabolism and tumor progression, highlighting the intricate involvement of arginine in the pathophysiology of liver cancer^82^. Lastly, OTC, which catalyzes the conversion of ornithine and carbamoyl phosphate into citrulline^72^, is crucial for ammonia detoxification. Deficiencies in OTC can lead to ammonia accumulation, a risk factor for HCC development^85,86^.

These metabolic shifts in the urea cycle and associated amino acid metabolism not only provide a deeper understanding of HCC pathogenesis, but also reveal potential targets for therapeutic intervention. Addressing the dysregulation of enzymes such as CPS1, ASS1, ARG1 and OTC could offer new strategies to curb HCC progression by manipulating the tumor’s metabolic dependencies.

#### BCAA metabolism

Branched-chain amino acids (BCAAs), which include leucine, isoleucine and valine, are essential amino acids distinguished by their nonlinear structure. In contrast to other amino acids, BCAAs are not endogenously produced by the human body and need to be obtained via the diet. These amino acids have a vital function in maintaining cell growth by serving as a nitrogen and/or carbon source^87^.

The isoenzymes BCAA aminotransferase 1 and 2 (BCAT1 and 2), which are located in the cytoplasm and mitochondria, respectively, convert BCAAs into the corresponding branched-chain keto acids (BCKAs) (Fig. 2d). BCKAs subsequently undergo oxidative decarboxylation catalyzed by the BCKA dehydrogenase (BCKDH) complex, resulting in the formation of acyl-coenzyme-A (acyl-CoA)^88^. The next steps of BCAA breakdown are similar to those of fatty acid oxidation (FAO), resulting in the formation of end products that can enter the TCA cycle.

The catabolism of isoleucine leads to the production of acetyl-CoA and propionyl-CoA, leucine produces acetyl-CoA and valine yields propionyl-CoA. These products are utilized by the TCA cycle, highlighting the role of BCAA catabolism in energy metabolism and cellular growth. This process is linked to cancer development, with BCAAs having a dual role of either promoting or inhibiting tumor growth depending on the genetic and tissue context^89–91^. Recent studies have shown that BCAAs act as signaling molecules that reflect the body’s nutritional state. As such, they can influence metabolic pathways related to cancer progression^92^ and increase the risk of HCC^93^. Also, enhanced breakdown of BCAAs in HCC and the redirection of their metabolic products toward nucleotide synthesis support rapid cell division and tumor growth. Furthermore, the expression of BCAT1 is elevated in HCC, correlating with increased tumor cell migration and invasion^94^. Conversely, long-term administration of BCAAs has been observed to improve the nutritional status and longevity of patients with liver cirrhosis, indicating a protective role under certain conditions^95–97^. BCAAs have demonstrated the ability to inhibit HCC caused by diethylnitrosamine in obese mice^98^ and rats^99^. Nevertheless, a decrease in the activity of BCAA catabolic enzymes results in elevated levels of BCAA and heightened activation of mTORC1, hence facilitating the growth of tumors^100^. Despite these insights, the complete mechanism by which BCAAs influence HCC development remains poorly understood and is a subject of ongoing research.

### Changes in FA metabolism

#### FA uptake and synthesis

The liver has an important role in mediating systemic lipid homeostasis, which includes the uptake, synthesis, storage, release and breakdown of fatty acids (FAs). FAs come from de novo synthesis within the liver or from the breakdown of fats stored in adipocytes during fasting or insulin resistance. Hepatocytes utilize fatty acid transporters (FATPs) and FA-binding proteins to facilitate FA uptake from the bloodstream. In the context of HCC, the metabolic processes of FA uptake and synthesis are significantly altered (Fig. 2d). Notably, the expression of FA transporters such as CD36 and FATP5 is upregulated in HCC, underscoring the importance of extracellular FA sources in cancer progression^101,102^. While normal cells predominantly rely on circulating exogenous FAs, HCC cells exhibit elevated de novo FA synthesis alongside uptake. This affects the process of membrane formation and pathways involved in signaling^103^.

The synthesis of FAs begins with the conversion of citrate to acetyl-CoA, catalyzed by ATP citrate lyase (ACLY). Subsequent reactions involving acetyl-CoA carboxylase (ACC) and fatty acid synthase (FASN) lead to the production of FAs. Stearoyl-CoA desaturase 1 (SCD1) then desaturates these FAs to produce monounsaturated FAs (MUFAs), crucial for triacylglycerol (TAG) synthesis^104^. The overexpression of enzymes such as ACLY^105^, ACC^105,106^, FASN^105,107^ and SCD1 (ref. ^105^) is commonly observed in HCC, enhancing lipid synthesis pathways and contributing to tumor growth.

AMP-activated protein kinase (AMPK) phosphorylates and thereby inhibits ACC, thus exerting an influence on hepatic de novo FA synthesis and potentially affecting carcinogenesis^108,109^. Consistent with this notion, a novel liver-specific ACC inhibitor, ND-654, mimics the effects of ACC phosphorylation, inhibiting hepatic de novo FA synthesis and HCC development^109^. Similarly, SCD1 which is involved in the synthesis of MUFAs, is associated with tumor proliferation in xenograft animal models and the survival rates of patients^27^. Although FASN does not directly transform hepatocytes into cancer cells, its role is crucial in the progression of hepatic steatosis and tumor development, particularly influenced by AKT signaling^107^. A preclinical study suggests that TVB3664, a novel FASN inhibitor, significantly ameliorates the fatty liver phenotype, although TVB3664 monotherapy shows moderate efficacy in metabolic dysfunction-associated steatohepatitis (MASH)-related murine HCCs^110^.

The transcription factor sterol regulatory element-binding protein-1 (SREBP-1) orchestrates the expression of FA synthesis genes, significantly impacting cellular proliferation^111^, migration^111^ and survival in HCC^112–114^. Dysregulation of SREBP-1, along with aberrant mTORC1 and mTORC2 signaling^79,115–118^, further complicates FA metabolic reprogramming in HCC. For instance, the deletion of hepatic tuberous sclerosis 1 (TSC1) a suppressor of mTORC1 signaling, has been demonstrated to stimulate mTORC1 and trigger the formation of spontaneous HCC with slight FA accumulation^119^. Notably, another study revealed similar cases of spontaneous HCC formation in mice lacking TSC1. However, in this particular case, the observed characteristics were ascribed to defects in the maturation of SREBP-1 and de novo FA synthesis, rather than direct activation of mTORC1 (refs. ^120,121^). The aforementioned results highlight the relationship between mTORC1 signaling, involving Akt, and lipid metabolism within the framework of hepatocarcinogenesis. It is worth noting that the activation of mTORC1 alone does not adequately stimulate hepatic SREBP-1. However, when rictor, a component of mTORC2, is specifically knocked out in the liver, there is a decrease in Akt Ser473 phosphorylation and a reduction in SREBP-1 activity, resulting in defective lipogenesis and hypolipidemia^118^. In addition, IGF-induced phosphorylation of Akt Ser473 leads to Akt-mediated phosphorylation of cytosolic phosphoenolpyruvate carboxykinase 1 (PCK1), the rate-limiting enzyme in gluconeogenesis. Phosphorylated PCK1 translocates to the endoplasmic reticulum, activating SREBP and enhancing the transcription of lipogenic genes by disrupting the interaction between INSIG proteins and SREBP cleavage-activating protein^122^. Thus, mTORC2 mediates hepatic lipid metabolism via insulin-induced Akt signaling. This underscores the complex interplay between the mTORC1 and mTORC2 pathways in the context of HCC progression. Furthermore, in animals lacking both tumor suppressors PTEN and TSC1, mTORC2 has been linked to the development of HCC, partly by activating SREBP-1 and facilitating FA production. This study provides a comprehensive understanding of the function of mTORC2 in HCC through its regulation of lipid metabolism, hence offering insights into prospective treatment strategies^123^.

#### FA breakdown

The liver possesses the capacity to metabolize FAs via both the mitochondrial and peroxisomal β-oxidation pathways (FAO). Carnitine palmitoyltransferase 1 (CPT1)^124^, essential for transporting FAs into mitochondria for oxidation, shows decreased activity in HCC contexts^125–127^ (Fig. 2d). This dysfunction contributes to the oncogenic potential of FA metabolism in HCC^55^. A thorough examination of metabolic profiles in obesity-induced HCC mouse models revealed a notable accumulation of long-chain acylcarnitine species, which is attributed to reduced conversion of acylcarnitine to acyl-CoA, probably due to reduced expression of CPT2. The downregulation of CPT2 leads to suppression of FAO, contributing to the steatotic changes commonly seen in HCC. Moreover, CPT2-mediated lipid metabolic reprogramming not only facilitates the adaptation of HCC cells to a lipid-rich microenvironment, but also drives liver tumor progression^128^. Previous studies have further demonstrated reduced CPT2 expression in patients with MASH-related HCC^128^, alongside elevated acylcarnitine levels in HCC mouse models and patients^129^. These findings suggest a potential metabolic shift that may contribute to the progression of obesity and MASH-related HCC. In addition, it is worth noting that medium-chain acyl-CoA dehydrogenase (MCAD) and long-chain CAD (LCAD), dehydrogenases involved in the initiation of FAO, display reduced expression in HCC tumors. MCAD has a role in the progress of HCC by interfering with the pathways involved in FAO, while the reduction in LCAD facilitates the progression of HCC by causing an accumulation of unsaturated FAs within cells^113,130^. The aforementioned findings underscore the relationship between the dysregulation of FAO and the development of HCC.

### Combining multiple metabolic reprogramming events

**Fig. 3:**
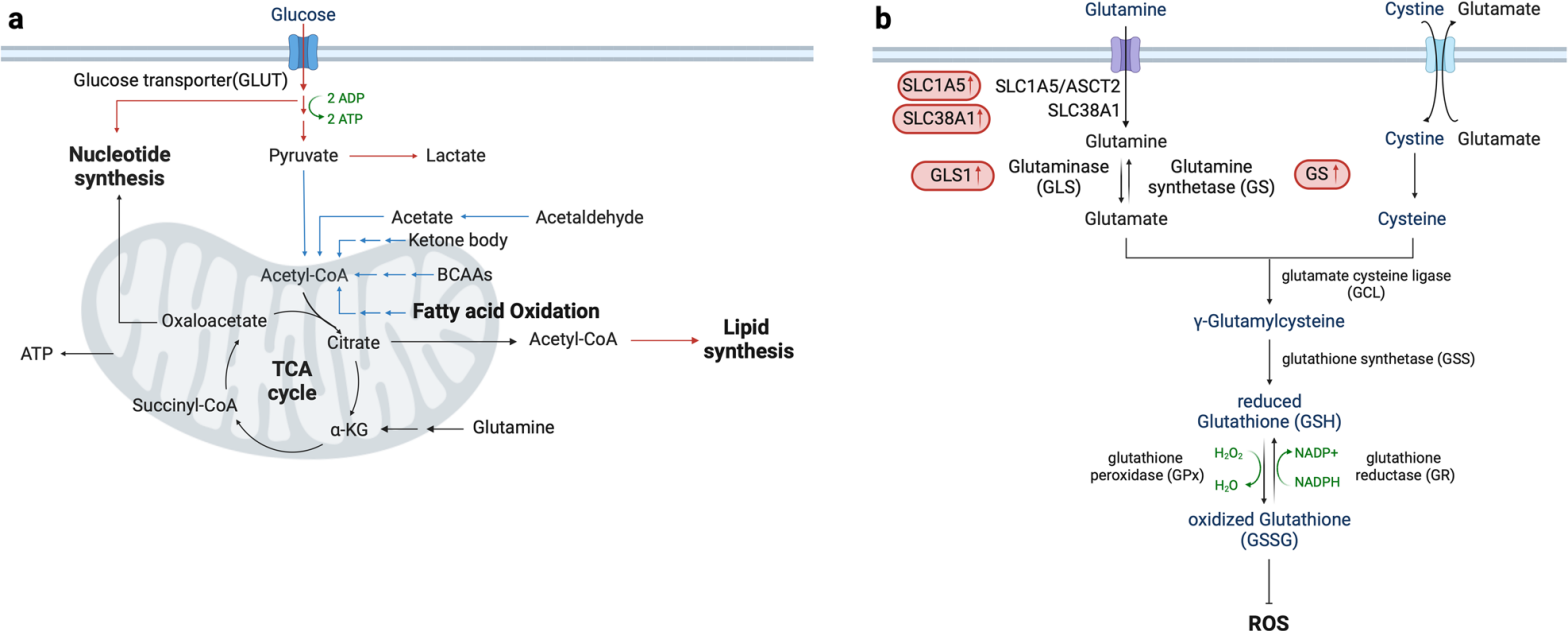
Rewired acetyl-CoA metabolism and redox homeostasis in HCC. The intricate interactions between various metabolic pathways that influence acetyl-CoA metabolism and redox homeostasis in HCC cells. **a**, Acetyl-CoA production is significantly reduced in HCC due to the downregulation of its biosynthetic pathways. This reduction impacts both energy metabolism and protein acetylation, subsequently affecting gene expression and cellular differentiation. **b**, HCC cells manage oxidative stress by enhancing antioxidant defenses, primarily through the glutathione–glutamine–glutamate pathway. Despite elevated levels of ROS, these adaptive mechanisms are essential for maintaining redox homeostasis, which is crucial for cancer cell viability, progression and recurrence. Upregulation of GLS1 in HCC increases glutamate production, thereby boosting antioxidant capacity and promoting characteristics associated with cancer stemness. Proteins and pathways that are upregulated in HCC are marked in red, while those that are downregulated are indicated in blue, providing a clear visual representation of the metabolic changes in HCC.

Metabolic reprogramming involves alterations across multiple biochemical pathways. One common aspect is a disturbance in acetyl-CoA metabolism, which is central to various physiological processes such as the TCA cycle, lipid synthesis and protein acetylation^131^. In addition to via breakdown of FAO and BCAA in cells, acetyl-CoA is also generated through the oxidative decarboxylation of pyruvate obtained from glycolysis^132^. In HCC, there is a noted reduction in acetyl-CoA production^133^ due to transcriptional downregulation of acetyl-CoA synthesis pathways, including FAO^128^, BCAA catabolism^100^ and pyruvate catabolism (Fig. 3a). This reduction impacts not only energy metabolism, but also the acetylation of histone and non-histone proteins, influencing gene expression and cellular differentiation. The downregulation of acetyl-CoA levels in HCC is mediated by the two transcription factors TEAD2 and E2A. These transcription factors, via downregulation of acetyl-CoA biosynthesis genes, drive proliferation and dedifferentiation in HCC, ultimately leading to poor patient survival^133^.

Oxidative stress is due to an imbalance of ROS production and antioxidant defenses^134–136^. Despite increased ROS, HCC cells maintain redox homeostasis through robust antioxidant mechanisms^135^, notably involving the glutathione–glutamine–glutamate pathway. In HCC, imported glutamine is converted to glutamate, which is a precursor for the synthesis of glutathione (Fig. 3b). Glutathione, a potent antioxidant, protects cancer cells from oxidative harm, thereby facilitating cellular viability and proliferation. The upregulation of GLS1 in HCC cells enhances glutamate production, which, in turn, increases antioxidant capacity and gene expression associated with stemness^137^. The maintenance of redox homeostasis is important for the progression and recurrence of HCC^138^, since oxidative damage enhances susceptibility to the illness^68^. Furthermore, HCC exhibits indications of DNA and lipid oxidative damage^139^, while patients with MASH-related HCC demonstrate decreased serum antioxidative function compared with patients with metabolic dysfunction-associated fatty liver disease^140^. These results collectively highlight the complex relationship between metabolic reprogramming and hepatocarcinogenesis, emphasizing the diverse functions of metabolism in promoting the development and progression of HCC.

## Conclusion

Oncogenic metabolic reprogramming is a dynamic and complex process influenced by numerous inputs responding to various cellular conditions. This reprogramming results in highly adaptable cellular states allowing survival and proliferation under diverse and challenging conditions. The interconnected nature of metabolism means that alterations in one pathway can significantly impact others, creating a network of metabolic changes that collectively contribute to cancer progression. Understanding the multilevel control of this reprogramming may reveal metabolic vulnerabilities, opening promising new avenues for therapeutic strategies. Such precision medicine approaches have the potential to enhance the effectiveness of treatments by directly targeting the unique metabolic dependencies of tumor cells.

## References

[1] Dayton, T. L. et al. Germline loss of PKM2 promotes metabolic distress and hepatocellular carcinoma. *Genes Dev.* **30**, 1020–1033 (2016). 27125672 10.1101/gad.278549.116 PMC4863734

[2] Ling, Z. N. et al. Amino acid metabolism in health and disease. *Signal. Transduct. Target. Ther.* **8**, 345 (2023). 10.1038/s41392-023-01569-3 PMC10497558 37699892

[3] Gluchowski, N. L., Becuwe, M., Walther, T. C. & Farese, R. V. Jr. Lipid droplets and liver disease: from basic biology to clinical implications. *Nat. Rev. Gastroenterol. Hepatol.* **14**, 343–355 (2017). 28428634 10.1038/nrgastro.2017.32 PMC6319657

[4] Liu, X., Wang, H., Liang, X. & Roberts, M. S. in *Liver Pathophysiology* 391–400 (Academic Press, 2017).

[5] Josep, M. et al. SHARP Investigators Study Group. Sorafenib in advanced hepatocellular carcinoma. *N. Engl. J. Med.* **359**, 378–390 (2008). 18650514 10.1056/NEJMoa0708857

[6] Cheng, A.-L. et al. Efficacy and safety of sorafenib in patients in the Asia–Pacific region with advanced hepatocellular carcinoma: a phase III randomised, double-blind, placebo-controlled trial. *Lancet Oncol.* **10**, 25–34 (2009). 19095497 10.1016/S1470-2045(08)70285-7

[7] Ikeda, M. et al. Safety and pharmacokinetics of lenvatinib in patients with advanced hepatocellular carcinoma. *Clin. Cancer Res.* **22**, 1385–1394 (2016). 26500236 10.1158/1078-0432.CCR-15-1354

[8] Abou-Alfa, G. K. et al. Cabozantinib in patients with advanced and progressing hepatocellular carcinoma. *N. Engl. J. Med.* **379**, 54–63 (2018). 29972759 10.1056/NEJMoa1717002 PMC7523244

[9] El-Khoueiry, A. B. et al. Nivolumab in patients with advanced hepatocellular carcinoma (CheckMate 040): an open-label, non-comparative, phase 1/2 dose escalation and expansion trial. *Lancet* **389**, 2492–2502 (2017). 28434648 10.1016/S0140-6736(17)31046-2 PMC7539326

[10] Kumari R., Sahu M. K., Tripathy A., Uthansingh K. & Behera M. Hepatocellular carcinoma treatment: hurdles, advances and prospects. *Hepat Oncol.*10.2217/hep-2018-0002 (2018). 10.2217/hep-2018-0002 PMC6613045 31293776

[11] Llovet, J. M. et al. Hepatocellular carcinoma. *Nat. Rev. Dis. Primers* **7**, 6 (2021). 33479224 10.1038/s41572-020-00240-3 PMC13537433

[12] Vander Heiden M. G., Cantley L. C. & Thompson C. B. Understanding the Warburg effect: the metabolic requirements of cell proliferation. *Science*10.1126/science.1160809 (2009). 10.1126/science.1160809 PMC2849637 19460998

[13] Macheda, M. L., Rogers, S. & Best, J. D. Molecular and cellular regulation of glucose transporter (GLUT) proteins in cancer. *J. Cell. Physiol.* **202**, 654–662 (2005). 15389572 10.1002/jcp.20166

[14] Amann, T. et al. GLUT1 expression is increased in hepatocellular carcinoma and promotes tumorigenesis. *Am. J. Pathol.* **174**, 1544–1552 (2009). 19286567 10.2353/ajpath.2009.080596 PMC2671384

[15] Sun, H. W. et al. GLUT1 and ASCT2 as predictors for prognosis of hepatocellular carcinoma. *PLoS ONE* **11**, e0168907 (2016). 28036362 10.1371/journal.pone.0168907 PMC5201247

[16] Kim, Y. H. et al. SLC2A2 (GLUT2) as a novel prognostic factor for hepatocellular carcinoma. *Oncotarget* **8**, 68381–68392 (2017). 28978124 10.18632/oncotarget.20266 PMC5620264

[17] Huang, Q. et al. Metabolic characterization of hepatocellular carcinoma using nontargeted tissue metabolomics. *Cancer Res.* **73**, 4992–5002 (2013). 23824744 10.1158/0008-5472.CAN-13-0308

[18] Robey, R. B. & Hay, N. Mitochondrial hexokinases, novel mediators of the antiapoptotic effects of growth factors and Akt. *Oncogene* **25**, 4683–4696 (2006). 16892082 10.1038/sj.onc.1209595

[19] DeWaal, D. et al. Hexokinase-2 depletion inhibits glycolysis and induces oxidative phosphorylation in hepatocellular carcinoma and sensitizes to metformin. *Nat. Commun.* **9**, 446 (2018). 29386513 10.1038/s41467-017-02733-4 PMC5792493

[20] Guzman, G. et al. Evidence for heightened hexokinase II immunoexpression in hepatocyte dysplasia and hepatocellular carcinoma. *Dig. Dis. Sci.* **60**, 420–426 (2015). 25381201 10.1007/s10620-014-3364-3 PMC4323170

[21] Xu, S. & Herschman, H. R. A tumor agnostic therapeutic strategy for hexokinase 1-null/hexokinase 2-positive cancers. *Cancer Res.* **79**, 5907–5914 (2019). 31434645 10.1158/0008-5472.CAN-19-1789 PMC12139393

[22] Arora, K. K. & Pedersen, P. L. Functional significance of mitochondrial bound hexokinase in tumor cell metabolism. Evidence for preferential phosphorylation of glucose by intramitochondrially generated ATP. *J. Biol. Chem.* **263**, 17422–17428 (1988). 3182854

[23] David, C. J., Chen, M., Assanah, M., Canoll, P. & Manley, J. L. HnRNP proteins controlled by c-Myc deregulate pyruvate kinase mRNA splicing in cancer. *Nature* **463**, 364–368 (2010). 20010808 10.1038/nature08697 PMC2950088

[24] Yu, G. et al. PKM2 regulates neural invasion of and predicts poor prognosis for human hilar cholangiocarcinoma. *Mol. Cancer* **14**, 193 (2015). 10.1186/s12943-015-0462-6 PMC4650283 26576639

[25] Chen, Z. et al. Co-expression of PKM2 and TRIM35 predicts survival and recurrence in hepatocellular carcinoma. *Oncotarget* **6**, 2538–2548 (2015). 25576919 10.18632/oncotarget.2991 PMC4385869

[26] Faloppi, L. et al. The role of LDH serum levels in predicting global outcome in HCC patients treated with sorafenib: implications for clinical management. *BMC Cancer* **14**, 110 (2014). 10.1186/1471-2407-14-110 PMC3930857 24552144

[27] Budhu, A. et al. Integrated metabolite and gene expression profiles identify lipid biomarkers associated with progression of hepatocellular carcinoma and patient outcomes. *Gastroenterology* **144**, 1066–1075.e1061 (2013). 23376425 10.1053/j.gastro.2013.01.054 PMC3633738

[28] Beyoglu, D. et al. Tissue metabolomics of hepatocellular carcinoma: tumor energy metabolism and the role of transcriptomic classification. *Hepatology* **58**, 229–238 (2013). 23463346 10.1002/hep.26350 PMC3695036

[29] He, S. & Tang, S. WNT/β-catenin signaling in the development of liver cancers. *Biomed. Pharmacother.* **132**, 110851 (2020). 33080466 10.1016/j.biopha.2020.110851

[30] Fan, H. et al. Critical role of mTOR in regulating aerobic glycolysis in carcinogenesis (review). *Int. J. Oncol.* **58**, 9–19 (2021). 10.3892/ijo.2020.5152 33367927

[31] Dentin, R. et al. Liver-specific inhibition of ChREBP improves hepatic steatosis and insulin resistance in ob/ob mice. *Diabetes* **55**, 2159–2170 (2006). 16873678 10.2337/db06-0200

[32] Zhang, L. et al. Glycolysis-related gene expression profiling serves as a novel prognosis risk predictor for human hepatocellular carcinoma. *Sci. Rep.* **11**, 18875 (2021). 34556750 10.1038/s41598-021-98381-2 PMC8460833

[33] Lee, N. C. W., Carella, M. A., Papa, S. & Bubici, C. High expression of glycolytic genes in cirrhosis correlates with the risk of developing liver cancer. *Front. Cell. Dev. Biol.* **6**, 138 (2018). 10.3389/fcell.2018.00138 PMC6220322 30430110

[34] Li, M. et al. Novel mitochondrion-targeting copper(II) complex induces HK2 malfunction and inhibits glycolysis via Drp1-mediating mitophagy in HCC. *J. Cell. Mol. Med.* **24**, 3091–3107 (2020). 31994339 10.1111/jcmm.14971 PMC7077532

[35] Li, M., Zhang, A., Qi, X., Yu, R. & Li, J. A novel inhibitor of PGK1 suppresses the aerobic glycolysis and proliferation of hepatocellular carcinoma. *Biomed. Pharmacother.* **158**, 114115 (2023). 36516697 10.1016/j.biopha.2022.114115

[36] Yao, J. et al. Isoginkgetin, a potential CDK6 inhibitor, suppresses SLC2A1/GLUT1 enhancer activity to induce AMPK–ULK1-mediated cytotoxic autophagy in hepatocellular carcinoma. *Autophagy* **19**, 1221–1238 (2023). 36048765 10.1080/15548627.2022.2119353 PMC10012924

[37] Ma, W. K. et al. ASO-based PKM splice-switching therapy inhibits hepatocellular carcinoma growth. *Cancer Res.* **82**, 900–915 (2022). 34921016 10.1158/0008-5472.CAN-20-0948 PMC8898261

[38] Li, M. et al. Aldolase B suppresses hepatocellular carcinogenesis by inhibiting G6PD and pentose phosphate pathways. *Nat. Cancer* **1**, 735–747 (2020). 35122041 10.1038/s43018-020-0086-7

[39] Wada, H. et al. Expression pattern of angiogenic factors and prognosis after hepatic resection in hepatocellular carcinoma: importance of angiopoietin-2 and hypoxia-induced factor-1 alpha. *Liver Int.* **26**, 414–423 (2006). 16629644 10.1111/j.1478-3231.2006.01243.x

[40] TeSlaa, T., Ralser, M., Fan, J. & Rabinowitz, J. D. The pentose phosphate pathway in health and disease. *Nat. Metab.* **5**, 1275–1289 (2023). 37612403 10.1038/s42255-023-00863-2 PMC11251397

[41] Patra, K. C. & Hay, N. The pentose phosphate pathway and cancer. *Trends Biochem. Sci.* **39**, 347–354 (2014). 25037503 10.1016/j.tibs.2014.06.005 PMC4329227

[42] Kruger, N. J. & von Schaewen, A. The oxidative pentose phosphate pathway: structure and organisation. *Curr. Opin. Plant Biol.* **6**, 236–246 (2003). 12753973 10.1016/s1369-5266(03)00039-6

[43] Uhlen, M. et al. A pathology atlas of the human cancer transcriptome. *Science* **357**, eaan2507 (2017). 28818916 10.1126/science.aan2507

[44] Nwosu, Z. C. et al. Identification of the consistently altered metabolic targets in human hepatocellular carcinoma. *Cell. Mol. Gastroenterol. Hepatol.* **4**, 303–323 e301 (2017). 28840186 10.1016/j.jcmgh.2017.05.004 PMC5560912

[45] Yin, X. et al. ID1 promotes hepatocellular carcinoma proliferation and confers chemoresistance to oxaliplatin by activating pentose phosphate pathway. *J. Exp. Clin. Cancer Res.* **36**, 166 (2017). 10.1186/s13046-017-0637-7 PMC5701377 29169374

[46] Lu, M. et al. Elevated G6PD expression contributes to migration and invasion of hepatocellular carcinoma cells by inducing epithelial–mesenchymal transition. *Acta Biochim. Biophys. Sin.* **50**, 370–380 (2018). 29471502 10.1093/abbs/gmy009

[47] Kowalik, M. A. et al. Metabolic reprogramming identifies the most aggressive lesions at early phases of hepatic carcinogenesis. *Oncotarget* **7**, 32375–32393 (2016). 27070090 10.18632/oncotarget.8632 PMC5078020

[48] Evert, M. et al. V-AKT murine thymoma viral oncogene homolog/mammalian target of rapamycin activation induces a module of metabolic changes contributing to growth in insulin-induced hepatocarcinogenesis. *Hepatology* **55**, 1473–1484 (2012). 22271091 10.1002/hep.25600

[49] Ngo, H. K. C., Kim, D. H., Cha, Y. N., Na, H. K. & Surh, Y. J. Nrf2 mutagenic activation drives hepatocarcinogenesis. *Cancer Res.* **77**, 4797–4808 (2017). 28655791 10.1158/0008-5472.CAN-16-3538

[50] Xu, I. M. et al. Transketolase counteracts oxidative stress to drive cancer development. *Proc. Natl Acad. Sci. USA* **113**, E725–E734 (2016). 26811478 10.1073/pnas.1508779113 PMC4760787

[51] Qin, Z. et al. Transketolase (TKT) activity and nuclear localization promote hepatocellular carcinoma in a metabolic and a non-metabolic manner. *J. Exp. Clin. Cancer Res.* **38**, 154 (2019). 10.1186/s13046-019-1131-1 PMC6458711 30971297

[52] Wise, D. R. & Thompson, C. B. Glutamine addiction: a new therapeutic target in cancer. *Trends Biochem. Sci.* **35**, 427–433 (2010). 20570523 10.1016/j.tibs.2010.05.003 PMC2917518

[53] Yang, L., Venneti, S. & Nagrath, D. Glutaminolysis: a hallmark of cancer metabolism. *Annu. Rev. Biomed. Eng.* **19**, 163–194 (2017). 28301735 10.1146/annurev-bioeng-071516-044546

[54] Yang, W. H., Qiu, Y., Stamatatos, O., Janowitz, T. & Lukey, M. J. Enhancing the efficacy of glutamine metabolism inhibitors in cancer therapy. *Trends Cancer* **7**, 790–804 (2021). 34020912 10.1016/j.trecan.2021.04.003 PMC9064286

[55] Bjornson, E. et al. Stratification of hepatocellular carcinoma patients based on acetate utilization. *Cell Rep.* **13**, 2014–2026 (2015). 26655911 10.1016/j.celrep.2015.10.045

[56] DeBerardinis, R. J., Lum, J. J., Hatzivassiliou, G. & Thompson, C. B. The biology of cancer: metabolic reprogramming fuels cell growth and proliferation. *Cell Metab.* **7**, 11–20 (2008). 18177721 10.1016/j.cmet.2007.10.002

[57] Zhu, W. W. et al. The fuel and engine: the roles of reprogrammed metabolism in metastasis of primary liver cancer. *Genes Dis.* **7**, 299–307 (2020). 32884984 10.1016/j.gendis.2020.01.016 PMC7452537

[58] Yu, D. et al. Kidney-type glutaminase (GLS1) is a biomarker for pathologic diagnosis and prognosis of hepatocellular carcinoma. *Oncotarget* **6**, 7619–7631 (2015). 25844758 10.18632/oncotarget.3196 PMC4480704

[59] Long, J. et al. Expression level of glutamine synthetase is increased in hepatocellular carcinoma and liver tissue with cirrhosis and chronic hepatitis B. *Hepatol. Int.* **5**, 698–706 (2011). 21484108 10.1007/s12072-010-9230-2 PMC3090553

[60] Liu, P. et al. Glutamine synthetase promotes tumor invasion in hepatocellular carcinoma through mediating epithelial–mesenchymal transition. *Hepatol. Res.* **50**, 246–257 (2020). 31652385 10.1111/hepr.13433

[61] Sohn, B. H., Park, I. Y., Shin, J. H., Yim, S. Y. & Lee, J. S. Glutamine synthetase mediates sorafenib sensitivity in β-catenin-active hepatocellular carcinoma cells. *Exp. Mol. Med.* **50**, e421 (2018). 29303508 10.1038/emm.2017.174 PMC5992988

[62] Tsujikawa, H. et al. Immunohistochemical molecular analysis indicates hepatocellular carcinoma subgroups that reflect tumor aggressiveness. *Hum. Pathol.* **50**, 24–33 (2016). 26997435 10.1016/j.humpath.2015.10.014

[63] Zhang, B. et al. Glutamine synthetase predicts adjuvant TACE response in hepatocellular carcinoma. *Int. J. Clin. Exp. Med.* **8**, 20722–20731 (2015). PMC4723840 26884995

[64] Adebayo Michael, A. O. et al. Inhibiting glutamine-dependent mTORC1 activation ameliorates liver cancers driven by β-catenin mutations. *Cell Metab.* **29**, 1135–1150.e1136 (2019). 30713111 10.1016/j.cmet.2019.01.002 PMC6506359

[65] Wang, Y. S. et al. Sirtuin 4 depletion promotes hepatocellular carcinoma tumorigenesis through regulating adenosine-monophosphate-activated protein kinase alpha/mammalian target of rapamycin axis in mice. *Hepatology* **69**, 1614–1631 (2019). 30552782 10.1002/hep.30421

[66] Wei, Y. et al. An RNA–RNA crosstalk network involving HMGB1 and RICTOR facilitates hepatocellular carcinoma tumorigenesis by promoting glutamine metabolism and impedes immunotherapy by PD-L1^+^ exosomes activity. *Signal. Transduct. Target. Ther.* **6**, 421 (2021). 34916485 10.1038/s41392-021-00801-2 PMC8677721

[67] Huang, X., Gan, G., Wang, X., Xu, T. & Xie, W. The HGF–MET axis coordinates liver cancer metabolism and autophagy for chemotherapeutic resistance. *Autophagy* **15**, 1258–1279 (2019). 30786811 10.1080/15548627.2019.1580105 PMC6613896

[68] Fitian, A. I. & Cabrera, R. Disease monitoring of hepatocellular carcinoma through metabolomics. *World J. Hepatol.* **9**, 1–17 (2017). 28105254 10.4254/wjh.v9.i1.1 PMC5220267

[69] Guo, H. et al. NRF2 SUMOylation promotes de novo serine synthesis and maintains HCC tumorigenesis. *Cancer Lett.* **466**, 39–48 (2019). 31546024 10.1016/j.canlet.2019.09.010

[70] Wang, K. et al. PHGDH arginine methylation by PRMT1 promotes serine synthesis and represents a therapeutic vulnerability in hepatocellular carcinoma. *Nat. Commun.* **14**, 1011 (2023). 36823188 10.1038/s41467-023-36708-5 PMC9950448

[71] Wei, L. et al. Genome-wide CRISPR–Cas9 library screening identified PHGDH as a critical driver for sorafenib resistance in HCC. *Nat. Commun.* **10**, 4681 (2019). 31615983 10.1038/s41467-019-12606-7 PMC6794322

[72] Watford, M. The urea cycle: teaching intermediary metabolism in a physiological setting. *Biochem. Mol. Biol. Educ.* **31**, 289–297 (2006).

[73] Tenen, D. G., Chai, L. & Tan, J. L. Metabolic alterations and vulnerabilities in hepatocellular carcinoma. *Gastroenterol. Rep.* **9**, 1–13 (2021). 10.1093/gastro/goaa066 PMC7962738 33747521

[74] McAlpine, J. A., Lu, H. T., Wu, K. C., Knowles, S. K. & Thomson, J. A. Down-regulation of argininosuccinate synthetase is associated with cisplatin resistance in hepatocellular carcinoma cell lines: implications for PEGylated arginine deiminase combination therapy. *BMC Cancer* **14**, 621 (2014). 10.1186/1471-2407-14-621 PMC4153943 25164070

[75] Hajaj, E., Sciacovelli, M., Frezza, C. & Erez, A. The context-specific roles of urea cycle enzymes in tumorigenesis. *Mol. Cell* **81**, 3749–3759 (2021). 34469752 10.1016/j.molcel.2021.08.005

[76] Tao, X. et al. Argininosuccinate synthase 1 suppresses cancer cell invasion by inhibiting STAT3 pathway in hepatocellular carcinoma. *Acta Biochim. Biophys. Sin.* **51**, 263–276 (2019). 30883650 10.1093/abbs/gmz005

[77] Munder, M. Arginase: an emerging key player in the mammalian immune system. *Br. J. Pharmacol.* **158**, 638–651 (2009). 19764983 10.1111/j.1476-5381.2009.00291.x PMC2765586

[78] Niu, F. et al. Arginase: an emerging and promising therapeutic target for cancer treatment. *Biomed. Pharmacother.* **149**, 112840 (2022). 35316752 10.1016/j.biopha.2022.112840

[79] Mossmann, D., Park, S. & Hall, M. N. mTOR signalling and cellular metabolism are mutual determinants in cancer. *Nat. Rev. Cancer* **18**, 744–757 (2018). 30425336 10.1038/s41568-018-0074-8

[80] Kishikawa, T. et al. Decreased miR122 in hepatocellular carcinoma leads to chemoresistance with increased arginine. *Oncotarget* **6**, 8339–8352 (2015). 25826076 10.18632/oncotarget.3234 PMC4480756

[81] Missiaen, R. et al. GCN2 inhibition sensitizes arginine-deprived hepatocellular carcinoma cells to senolytic treatment. *Cell Metab*. **34**, 1151–1167.e1157 (2022). 35839757 10.1016/j.cmet.2022.06.010 PMC9357184

[82] Mossmann, D. et al. Arginine reprograms metabolism in liver cancer via RBM39. *Cell* **186**, 5068–5083.e5023 (2023). 37804830 10.1016/j.cell.2023.09.011 PMC10642370

[83] Richards, N. G. & Schuster, S. M. Mechanistic issues in asparagine synthetase catalysis. *Adv. Enzymol. Relat. Areas Mol. Biol.*10.1002/9780470123188.ch5 (1998). 10.1002/9780470123188.ch5 9559053

[84] Zhang, B. et al. Asparagine synthetase is an independent predictor of surgical survival and a potential therapeutic target in hepatocellular carcinoma. *Br. J. Cancer* **109**, 14–23 (2013). 23764751 10.1038/bjc.2013.293 PMC3708586

[85] Laemmle, A. et al. Frequency and pathophysiology of acute liver failure in ornithine transcarbamylase deficiency (OTCD). *PLoS ONE* **11**, e0153358 (2016). 27070778 10.1371/journal.pone.0153358 PMC4829252

[86] Wang, L. et al. AAV gene therapy corrects OTC deficiency and prevents liver fibrosis in aged OTC-knock out heterozygous mice. *Mol. Genet. Metab.* **120**, 299–305 (2017). 28283349 10.1016/j.ymgme.2017.02.011 PMC5423267

[87] Peng, H., Wang, Y. & Luo, W. Multifaceted role of branched-chain amino acid metabolism in cancer. *Oncogene* **39**, 6747–6756 (2020). 32978521 10.1038/s41388-020-01480-z PMC7606751

[88] Harper, E., Miller, R. H. & Block, K. P. Branched-chain amino acid metabolism. *Metabolism*10.1146/annurev.nu.04.070184.002205 (1984). 10.1146/annurev.nu.04.070184.002205 6380539

[89] Hattori, A. et al. Cancer progression by reprogrammed BCAA metabolism in myeloid leukaemia. *Nature* **545**, 500–504 (2017). 28514443 10.1038/nature22314 PMC5554449

[90] Li, J. T. et al. BCAT2-mediated BCAA catabolism is critical for development of pancreatic ductal adenocarcinoma. *Nat. Cell Biol.* **22**, 167–174 (2020). 32029896 10.1038/s41556-019-0455-6

[91] Mayers, J. R. et al. Tissue of origin dictates branched-chain amino acid metabolism in mutant Kras-driven cancers. *Science* **353**, 1161–1165 (2016). 27609895 10.1126/science.aaf5171 PMC5245791

[92] Suryawan, A. et al. A molecular model of human branched-chain amino acid metabolism. *Am. J. Clin. Nutr.* **68**, 72–81 (1998). 9665099 10.1093/ajcn/68.1.72

[93] Yang, D. et al. Branched-chain amino acid catabolism breaks glutamine addiction to sustain hepatocellular carcinoma progression. *Cell Rep.* **41**, 111691 (2022). 36417878 10.1016/j.celrep.2022.111691

[94] Xu, M. et al. BCAT1 promotes tumor cell migration and invasion in hepatocellular carcinoma. *Oncol. Lett.* **12**, 2648–2656 (2016). 27698837 10.3892/ol.2016.4969 PMC5038498

[95] Takegoshi, K. et al. Branched-chain amino acids prevent hepatic fibrosis and development of hepatocellular carcinoma in a non-alcoholic steatohepatitis mouse model. *Oncotarget*10.18632/oncotarget.15304 (2017). 10.18632/oncotarget.15304 PMC5392319 28212548

[96] Plauth, M. et al. ESPEN guideline on clinical nutrition in liver disease. *Clin. Nutr.* **38**, 485–521 (2019). 30712783 10.1016/j.clnu.2018.12.022 PMC6686849

[97] Kikuchi, Y. et al. A randomized clinical trial of preoperative administration of branched-chain amino acids to prevent postoperative ascites in patients with liver resection for hepatocellular carcinoma. *Ann. Surg. Oncol.* **23**, 3727–3735 (2016). 27338747 10.1245/s10434-016-5348-3

[98] Iwasa, J. et al. Dietary supplementation with branched-chain amino acids suppresses diethylnitrosamine-induced liver tumorigenesis in obese and diabetic C57BL/KsJ-db/db mice. *Cancer Sci.* **101**, 460–467 (2010). 19906067 10.1111/j.1349-7006.2009.01402.x PMC11159020

[99] Yoshiji, H. et al. Branched-chain amino acids suppress insulin-resistance-based hepatocarcinogenesis in obese diabetic rats. *J. Gastroenterol.* **44**, 483–491 (2009). 19319465 10.1007/s00535-009-0031-0

[100] Ericksen, R. E. et al. Loss of BCAA catabolism during carcinogenesis enhances mTORC1 activity and promotes tumor development and progression. *Cell Metab.* **29**, 1151–1165e1156 (2019). 30661928 10.1016/j.cmet.2018.12.020 PMC6506390

[101] Nelson, M. E. et al. Inhibition of hepatic lipogenesis enhances liver tumorigenesis by increasing antioxidant defence and promoting cell survival. *Nat. Commun.* **8**, 14689 (2017). 28290443 10.1038/ncomms14689 PMC5424065

[102] Gao, Q. et al. SLC27A5 deficiency activates NRF2/TXNRD1 pathway by increased lipid peroxidation in HCC. *Cell Death Differ.* **27**, 1086–1104 (2020). 31367013 10.1038/s41418-019-0399-1 PMC7206086

[103] Paul, B., Lewinska, M. & Andersen, J. B. Lipid alterations in chronic liver disease and liver cancer. *JHEP Rep.* **4**, 100479 (2022). 35469167 10.1016/j.jhepr.2022.100479 PMC9034302

[104] Currie, E., Schulze, A., Zechner, R., Walther, T. C. & Farese, R. V. Jr. Cellular fatty acid metabolism and cancer. *Cell Metab.* **18**, 153–161 (2013). 23791484 10.1016/j.cmet.2013.05.017 PMC3742569

[105] Calvisi, D. F. et al. Increased lipogenesis, induced by AKT–mTORC1–RPS6 signaling, promotes development of human hepatocellular carcinoma. *Gastroenterology* **140**, 1071–1083 (2011). 21147110 10.1053/j.gastro.2010.12.006 PMC3057329

[106] Wang, M. D. et al. Acetyl-coenzyme A carboxylase alpha promotion of glucose-mediated fatty acid synthesis enhances survival of hepatocellular carcinoma in mice and patients. *Hepatology* **63**, 1272–1286 (2016). 26698170 10.1002/hep.28415

[107] Li, L. et al. Inactivation of fatty acid synthase impairs hepatocarcinogenesis driven by AKT in mice and humans. *J. Hepatol.* **64**, 333–341 (2016). 26476289 10.1016/j.jhep.2015.10.004 PMC4718802

[108] Fullerton, M. D. et al. Single phosphorylation sites in Acc1 and Acc2 regulate lipid homeostasis and the insulin-sensitizing effects of metformin. *Nat. Med.* **19**, 1649–1654 (2013). 24185692 10.1038/nm.3372 PMC4965268

[109] Lally, J. S. V. et al. Inhibition of acetyl-CoA carboxylase by phosphorylation or the inhibitor ND-654 suppresses lipogenesis and hepatocellular carcinoma. *Cell Metab.* **29**, 174–182.e175 (2019). 30244972 10.1016/j.cmet.2018.08.020 PMC6643297

[110] Wang, H. et al. Therapeutic efficacy of FASN inhibition in preclinical models of HCC. *Hepatology* **76**, 951–966 (2022). 35076948 10.1002/hep.32359 PMC9309180

[111] Shao, W. & Espenshade, P. J. Expanding roles for SREBP in metabolism. *Cell Metab.* **16**, 414–419 (2012). 23000402 10.1016/j.cmet.2012.09.002 PMC3466394

[112] Li, C. et al. SREBP-1 has a prognostic role and contributes to invasion and metastasis in human hepatocellular carcinoma. *Int. J. Mol. Sci.* **15**, 7124–7138 (2014). 24776759 10.3390/ijms15057124 PMC4057663

[113] Ma, A. P. Y. et al. Suppression of ACADM-mediated fatty acid oxidation promotes hepatocellular carcinoma via aberrant CAV1/SREBP1 signaling. *Cancer Res.* **81**, 3679–3692 (2021). 33975883 10.1158/0008-5472.CAN-20-3944

[114] Yamashita, T. et al. Activation of lipogenic pathway correlates with cell proliferation and poor prognosis in hepatocellular carcinoma. *J. Hepatol.* **50**, 100–110 (2009). 19008011 10.1016/j.jhep.2008.07.036

[115] Porstmann, T. et al. SREBP activity is regulated by mTORC1 and contributes to Akt-dependent cell growth. *Cell Metab.* **8**, 224–236 (2008). 18762023 10.1016/j.cmet.2008.07.007 PMC2593919

[116] Li, S., Oh, Y. T., Yue, P., Khuri, F. R. & Sun, S. Y. Inhibition of mTOR complex 2 induces GSK3/FBXW7-dependent degradation of sterol regulatory element-binding protein 1 (SREBP1) and suppresses lipogenesis in cancer cells. *Oncogene* **35**, 642–650 (2016). 25893295 10.1038/onc.2015.123 PMC4615269

[117] Owen, J. L. et al. Insulin stimulation of SREBP-1c processing in transgenic rat hepatocytes requires p70 S6-kinase. *Proc. Natl Acad. Sci. USA* **109**, 16184–16189 (2012). 22927400 10.1073/pnas.1213343109 PMC3479583

[118] Hagiwara, A. et al. Hepatic mTORC2 activates glycolysis and lipogenesis through Akt, glucokinase, and SREBP1c. *Cell Metab.* **15**, 725–738 (2012). 22521878 10.1016/j.cmet.2012.03.015

[119] Li, T. et al. mTOR direct crosstalk with STAT5 promotes de novo lipid synthesis and induces hepatocellular carcinoma. *Cell Death Dis.* **10**, 619 (2019). 31409773 10.1038/s41419-019-1828-2 PMC6692326

[120] Yecies, J. L. et al. Akt stimulates hepatic SREBP1c and lipogenesis through parallel mTORC1-dependent and independent pathways. *Cell Metab.* **14**, 21–32 (2011). 21723501 10.1016/j.cmet.2011.06.002 PMC3652544

[121] Suchithra, M. et al. Chronic activation of mTOR complex 1 is sufficient to cause hepatocellular carcinoma in mice. *Sci. Signal.*10.1126/scisignal.200273 (2012). 10.1126/scisignal.2002739 PMC3743103 22457330

[122] Xu, D. et al. The gluconeogenic enzyme PCK1 phosphorylates INSIG1/2 for lipogenesis. *Nature* **580**, 530–535 (2020). 32322062 10.1038/s41586-020-2183-2

[123] Guri, Y. et al. mTORC2 promotes tumorigenesis via lipid synthesis. *Cancer Cell* **32**, 807–823.e812 (2017). 29232555 10.1016/j.ccell.2017.11.011

[124] Kerner, J. & Hoppel, C. Fatty acid import into mitochondria. *Biochim. Biophys. Acta*10.1016/S1388-1981(00)00044-5 (2000). 10.1016/s1388-1981(00)00044-5 10856709

[125] Serviddio, G. et al. Alterations of hepatic ATP homeostasis and respiratory chain during development of non-alcoholic steatohepatitis in a rodent model. *Eur. J. Clin. Invest.* **38**, 245–252 (2008). 18339004 10.1111/j.1365-2362.2008.01936.x

[126] Serviddio, G. et al. Oxidation of hepatic carnitine palmitoyl transferase-I (CPT-I) impairs fatty acid β-oxidation in rats fed a methionine-choline deficient diet. *PLoS ONE* **6**, e24084 (2011). 21909411 10.1371/journal.pone.0024084 PMC3164715

[127] Liu, Y. et al. CPT1A loss disrupts BCAA metabolism to confer therapeutic vulnerability in TP53-mutated liver cancer. *Cancer Lett*. 10.1016/j.canlet.2024.217006 (2024). 10.1016/j.canlet.2024.217006 38823763

[128] Fujiwara, N. et al. CPT2 downregulation adapts HCC to lipid-rich environment and promotes carcinogenesis via acylcarnitine accumulation in obesity. *Gut* **67**, 1493–1504 (2018). 29437870 10.1136/gutjnl-2017-315193 PMC6039238

[129] Yaligar, J. et al. Longitudinal metabolic imaging of hepatocellular carcinoma in transgenic mouse models identifies acylcarnitine as a potential biomarker for early detection. *Sci. Rep.* **6**, 20299 (2016). 26831370 10.1038/srep20299 PMC4735819

[130] Huang, D. et al. HIF-1-mediated suppression of acyl-CoA dehydrogenases and fatty acid oxidation is critical for cancer progression. *Cell Rep.* **8**, 1930–1942 (2014). 25242319 10.1016/j.celrep.2014.08.028

[131] Sivanand, S., Viney, I. & Wellen, K. E. Spatiotemporal control of acetyl-CoA metabolism in chromatin regulation. *Trends Biochem. Sci.* **43**, 61–74 (2018). 29174173 10.1016/j.tibs.2017.11.004 PMC5741483

[132] Pietrocola, F., Galluzzi, L., Bravo-San Pedro, J. M., Madeo, F. & Kroemer, G. Acetyl coenzyme A: a central metabolite and second messenger. *Cell Metab.* **21**, 805–821 (2015). 26039447 10.1016/j.cmet.2015.05.014

[133] Park, S. et al. Transcription factors TEAD2 and E2A globally repress acetyl-CoA synthesis to promote tumorigenesis. *Mol. Cell* **82**, 4246–4261.e4211 (2022). 36400009 10.1016/j.molcel.2022.10.027

[134] Muriel, P. Role of free radicals in liver diseases. *Hepatol. Int.* **3**, 526–536 (2009). 19941170 10.1007/s12072-009-9158-6 PMC2790593

[135] Sosa, V. et al. Oxidative stress and cancer: an overview. *Ageing Res. Rev.* **12**, 376–390 (2013). 23123177 10.1016/j.arr.2012.10.004

[136] Kudo, Y. et al. PKCλ/ι loss induces autophagy, oxidative phosphorylation, and NRF2 to promote liver cancer progression. *Cancer Cell* **38**, 247–262.e211 (2020). 32589943 10.1016/j.ccell.2020.05.018 PMC7423690

[137] Li, B. et al. Targeting glutaminase 1 attenuates stemness properties in hepatocellular carcinoma by increasing reactive oxygen species and suppressing Wnt/beta-catenin pathway. *EBioMedicine* **39**, 239–254 (2019). 30555042 10.1016/j.ebiom.2018.11.063 PMC6355660

[138] Kim, M. J. et al. PPARδ reprograms glutamine metabolism in sorafenib-resistant HCC. *Mol. Cancer Res.* **15**, 1230–1242 (2017). 28584024 10.1158/1541-7786.MCR-17-0061

[139] Tanaka, S. et al. Increased hepatic oxidative DNA damage in patients with nonalcoholic steatohepatitis who develop hepatocellular carcinoma. *J. Gastroenterol*. **48**, 1249–1258 (2013). 23329365 10.1007/s00535-012-0739-0

[140] Shimomura, Y. et al. The serum oxidative/anti-oxidative stress balance becomes dysregulated in patients with non-alcoholic steatohepatitis associated with hepatocellular carcinoma. *Intern. Med.* **56**, 243–251 (2017). 28154266 10.2169/internalmedicine.56.7002 PMC5348446

